# OTUD6B-AS1 Inhibits Viability, Migration, and Invasion of Thyroid Carcinoma by Targeting miR-183-5p and miR-21

**DOI:** 10.3389/fendo.2020.00136

**Published:** 2020-03-17

**Authors:** Zhuolu Wang, Fada Xia, Tiecheng Feng, Bo Jiang, Wenlong Wang, Xinying Li

**Affiliations:** Department of General Surgery, Xiangya Hospital, Central South University, Changsha, China

**Keywords:** lncRNA, OTUD6B-AS1, microRNA, thyroid carcinoma, miR-183-5p, miR-21

## Abstract

**Background:** The long noncoding RNA (lncRNA) functions as a regulator of initiation, progression, and metastasis of thyroid carcinomas. lncRNA OTUD6B antisense RNA 1 (OTUD6B-AS1) is a tumor-suppressive noncoding RNA in clear cell renal cell carcinoma. The role of OTUD6B-AS1 in thyroid carcinomas has not been reported yet. We aim to investigate the expression and biological functions of OTUD6B-AS1 in thyroid carcinomas.

**Methods:** The expression level of OTUD6B-AS1 was measured in 60 paired human thyroid carcinoma tissues and corresponding adjacent normal thyroid tissues. The correlations between the OTUD6B-AS1 expression levels and clinicopathological features were evaluated using the Mann-Whitney test. The effects of OTUD6B-AS1 on thyroid carcinoma cells were determined via the MTT and transwell assays. The potential targets of OTUD6B-AS1 were screened using the online programs OncomiR and StarBase 3.0, and the LncBase Predicted v.2. Luciferase reporter assay was used to confirm the interactions between OTUD6B-AS1 and its potential targets.

**Results:** OTUD6B-AS1 was downregulated in thyroid carcinoma tissue samples. The expression of OTUD6B-AS1 correlated with tumor size, clinical stage, and lymphatic metastasis of thyroid carcinoma. Overexpression of OTUD6B-AS1 significantly decreased the viability, migration, and invasion of thyroid carcinoma cells. Online programs predicted miR-183-5p and miR-21 as potential targets of OTUD6B-AS1. Luciferase reporter assays showed miR-183-5p and miR-21 bound to OTUD6B-AS1. Moreover, overexpression of miR-183-5p and miR-21 compromised the inhibitory effects of OTUD6B-AS1 on viability, migration, and invasion of thyroid carcinoma cells.

**Conclusions:** Taken together, our findings present *in vitro* evidence of lncRNA OTUD6B-AS1 as a tumor suppressor in thyroid carcinomas. OTUD6B-AS1 inhibits viability, migration, and invasion of thyroid carcinoma by targeting miR-183-5p and miR-21.

## Introduction

Thyroid carcinoma is a malignant tumor derived from thyroid epithelial cells (1). It exhibits multicentricity in the thyroid gland and frequently metastasizes to the regional lymph nodes (2). It can damage liver and kidney function and the respiratory system (2). Although some achievements have been made in the study of thyroid cancer, its pathogenesis is still unclear.

Long noncoding RNAs (lncRNAs) refer to the type of noncoding RNA that is derived from genome-encoded transcripts with lengths longer than 200 RNA nucleotides (3). Recent studies suggest it may be directly involved in the regulation of development of tumor cells (4–6). LncRNAs are involved in the regulatory network of thyroid carcinomas. Several lncRNAs have been reported to promote or inhibit proliferation, migration, invasion, and angiogenesis of thyroid carcinomas (7, 8). In addition, dysregulation of lncRNAs is associated with poor prognoses thyroid carcinomas (9). lncRNA OTUD6B antisense RNA 1 (OTUD6B-AS1) is oriented in an antisense direction relative to the protein-coding gene OTUD6B on the opposite DNA strand. OTUD6B-AS1 locates on chromosome 8q21.3 and has 2179 bp (NR_110439, ENST00000524003.1). OTUD6B-AS1 was initially reported as active in fibrosis in systemic sclerosis (10). It is downregulated in skin biopsies of systemic sclerosis and regulates the apoptosis of dermal fibroblasts (10). The role of OTUD6B-AS1 in cancer is largely unknown. A recent study reveals the role of OTUD6B-AS1 in clear cell renal cell carcinoma (ccRCC) (11). This study demonstrates that the lower expression of OTUD6B-AS1 is associated with poor prognosis of ccRCC (11). *In vitro* and *in vivo* evidence indicates that OTUD6B-AS1 functions as a tumor suppressor in ccRCC (11). However, the expressions and biological functions of OTUD6B-AS1 are still unknown in thyroid carcinomas.

microRNAs (miRNAs) have emerged as key post-transcriptional regulators of tumourigenesis in thyroid carcinoma which able to bind to the 3′-untranslated region of target mRNAs and inhibited the protein expression of target gene (12, 13). Additionally, miRNA can be sponged by lncRNAs via competitive endogenous RNA (ceRNA) mechanism to regulate the abundance of miRNAs, then regulate miRNAs targets expression (14). In thyroid carcinoma, lncRNA FOXD2-AS1 promoted the tumorigenesis via sponging miR-7-5p to upregulate TERT expression (15). Currently, no OTUD6B-AS1 that act as ceRNAs have been reported in thyroid carcinomas.

In this work, we investigated the expression of OTUD6B-AS1 in human thyroid carcinoma tissues and corresponding adjacent normal thyroid tissue samples. We also evaluated the correlations between the expression levels of OTUD6B-AS1 and the clinicopathological parameters. Moreover, we screened the potential targets of OTUD6B-AS1 and explored the ceRNAs molecular mechanisms underlying the regulatory effects of OTUD6B-AS1 on thyroid carcinoma cells.

## Materials and Methods

### Specimens

Sixty thyroid carcinoma tissues (include 1 undifferentiated thyroid carcinoma, 3 follicular thyroid carcinoma, and 56 papillary thyroid carcinoma) and the corresponding normal tissues were collected from patients undergoing thyroidectomy at the Xiangya Hospital. This study obtained approval from the Medical Ethics Committee of Xiangya Hospital, Central South University (Approval number: 201612010). Informed written consent for the use of the tissue samples was obtained from all the patients. Clinicopathological data of the patients were showed in Table 1. All fresh tissues were frozen in liquid nitrogen immediately and stored at −80°C until use.

**Table 1:** Clinicopathological data of the patients.

**Parameters**	**Groups**	**Numbers (*n* = 60)**
Age	≤45	32
	>45	28
Gender	Male	22
	Female	38
Multifocality	No	33
	Yes	27
Tumor size	<2	28
	≥2	32
Lymphatic metastasis	No	36
	Yes	24
TNM stage	I+II	38
	III+IV	22

### Cell Culture and Transfection

Thyroid carcinoma cells SW579, Papillary thyroid cancer cells TPC-1, and normal thyroid cells Nthy-ori were obtained from the American Type Culture Collection. Nthy-ori cells and TPC-1 cells were cultured in DMEM Medium (Gibco, Carlsbad, CA, USA) containing 10% fetal bovine serum (HyClone). SW579 cells were cultured in Leibovitz's L15 Medium (Gibco) containing 10% fetal bovine serum (HyClone). Full-length OTUD6B-AS1 was synthesized and sub-cloned into the pcDNA3.1 vector (OTUD6B-AS1 group); the empty pcDNA3.1 vector acts as a negative control (vector group). The mimics of miR-183-5p and miR-21 were obtained from GenePharma Co., Ltd. (Shanghai, China). The mimics were transfected into thyroid carcinoma cells using lipofectamine 2000 reagent (Invitrogen, Carlsbad, CA, USA) according to the manufacturer's instructions. The negative control mimic (NC mimic) was used as control. The transfection efficiency was determined by increased OTUD6B-AS1, miR-183-5p, and miR-21 expression using quantitative reverse transcription polymerase chain reaction (qRT-PCR).

### qRT-PCR

Total RNA was extracted using TRIzol reagent (Invitrogen) according to the manufacturer's instructions. Complementary DNA (cDNA) was used to determine the expression of OTUD6B-AS1 synthesized using a PrimeScript™ RT Reagent Kit (TaKaRa, Dalian, China) according to the manufacturer's instructions. The expression of OTUD6B-AS1, miR-183-5p, and miR-21 were determined using a SYBR® Premix Ex Taq™ II kit (TaKaRa), and18srRNA and U6 served as an internal reference. All the experiments were performed in duplicate. The results are represented as the fold-induction using the 2-ΔΔCT method (16). The primers used to determine the expression of OTUD6B-AS1 (11), miR-183-5p, and miR-21 are listed in Table 2.

**Table 2:** qRT-PCR primer.

**Gene**	**Primer sequence (5^′^ to 3^′^)**
OTUD6B-AS1	F: GACATATCCGGGTGACGTTTTT
	R: TTGTTCCACTGTCTTCTGGCATT
18srRNA	F: CCTGGATACCGCAGCTAGGA
	R: GCGGCGCAATACGAATGCCCC
hsa-miR-183-5p	F: ACACTCCAGCTGGGTATGGCACTGGTAGAA
	R: CTCAACTGGTGTCGTGGA
miR-21	F: ACACTCCAGCTGGGTAGCTTATCAGACTGA
	R: CTCAACTGGTGTCGTGGA
U6	F: CTCGCTTCGGCAGCACA
	R: AACGCTTCACGAATTTGCGT

### Cell Viability Assay

Cell proliferation was assayed using an MTT assay at 48 h after transfected. The absorbance of the solution was measured with a spectrophotometer at 490 nm.

### Transwell Assay

The transwell assay for migration was performed using chambers with polycarbonate filters (pore size 8μm) (Becton Dickinson Labware). The transwell assay for invasion was performed using chambers with Matrigel. Briefly, we harvested SW579 and TPC-1 cells and placed 5 × 10^4^ transfected cells in the upper chamber of the filter. After 24 h of incubation and removal of the cells in the upper chamber using a cotton swab, the cells on the underside were fixed with 4% paraformaldehyde, stained with 0.1% crystal violet in 20% ethanol, and counted in five randomly selected fields under a phase-contrast microscope. The migrated or invasive cells were monitored by photographing at 400× magnification using a LEICA microscope. The assays were performed in triplicate.

### *In silico* Prediction of OTUD6B-AS1 Potential Targets

To investigate the mechanisms underlying the inhibitory effect of OTUD6B-AS1 on thyroid carcinoma cells, we screened potential targets of OTUD6B-AS1 using several online programs, OncomiR (17), StarBase 3.0 (18), and LncBase Predicted v.2 (19). StarBase 3.0 and LncBase Predicted v.2 were used to predict the bind sites between OTUD6B-AS1 and miRNAs according to the keyword “OTUD6B-AS1”. Additionally, based on the tumor suppressive behavior of OTUD6B-AS1, we speculated that its targets may be cancer-promoting miRNAs. In OncomiR website, we search upregulated miRNAs in thyroid carcinoma by cancer type for significant miRNAs in tumor formation. The intersection of 3 results had analyzed by the Veen graph. Additionally, target mRNAs of miR-21 and miR-183-3p were analyzed by Targetscan 7.2 (20). KEGG pathway enrichment analysis of target mRNAs were used DAVID website (21).

### Luciferase Reporter Assays

Cells were seeded in 24-well plates. Cells were co-transfected with 150 ng of OTUD6B-AS1 reporter vector, 10 nM of miRNAs, were harvested 24 h after transfection, and luminescence was measured in triplicate using Promega Dual-Glo luciferase assay reagents according to the manufacturer's protocol, using an Optima series luminometer.

### Statistical Analysis

Statistical data were analyzed using the Statistical Program for Social Sciences (SPSS) 19.0 software (SPSS, Chicago, IL, USA). GraphPad Prism 7.0 (GraphPad Software, La Jolla, CA) was used to plot all the graphs. The differences between thyroid carcinoma tissue and adjacent normal tissues were analyzed using the Mann-Whitney test. An analysis of variance (ANOVA) was used for the overall comparison between three groups followed by the Newman-Keuls *t*-test used for multiple comparisons between the two groups. The correlations between the OTUD6B-AS1 expression and clinicopathological features were evaluated using the Mann-Whitney test. *P* < 0.05 was considered statistically significant.

## Results

### OTUD6B-AS1 Is Downregulated in Thyroid Carcinoma

**Figure 1:**
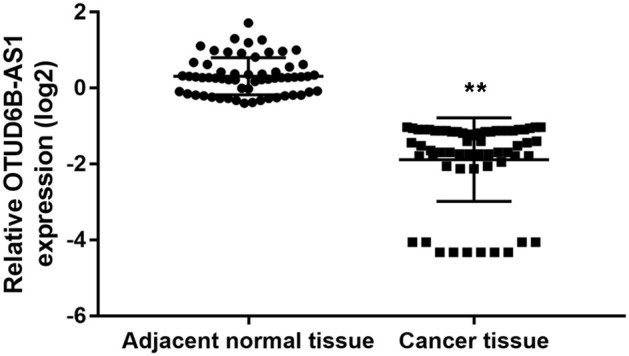
OTUD6B-AS1 is downregulated in thyroid carcinoma tissues. The expressions of OTUD6B-AS1 in 60 pairs of thyroid carcinoma tissues and adjacent para-cancerous tissues were evaluated using qRT-PCR. The correlations between the OTUD6B-AS1 expression and clinicopathological features were evaluated using the Mann-Whitney test. **p < 0.01.

**Figure 2:**
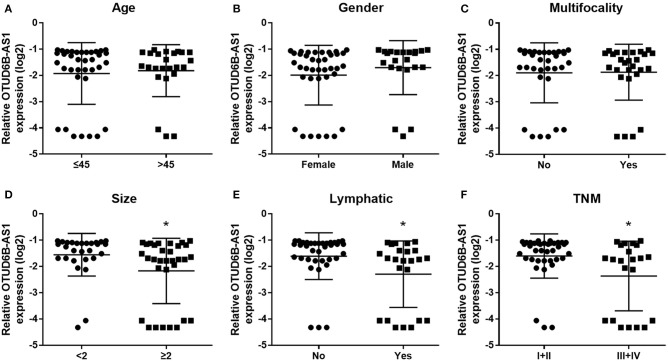
The correlations between the OTUD6B-AS1 expression and clinicopathological features were evaluated. The correlations between the OTUD6B-AS1 expression level and clinicopathological features were evaluated using the Mann-Whitney test. No correlation was observed between expression of OTUD6B-AS1 and age **(A)**, gender **(B)** and multifocality **(C)**. OTUD6B-AS1 correlated with tumor size **(D)**, lymphatic metastasis **(E)**, and clinical stage **(F)**. *p < 0.05.

We investigated the expression of OTUD6B-AS1 in 60 pair of thyroid carcinoma tissues and adjacent para-cancerous tissues. We found that OTUD6B-AS1 was downregulated in the thyroid carcinoma tissues compared to the para-cancerous tissues (*p* < 0.01, Figure 1). The correlations between the OTUD6B-AS1 expression level and the clinicopathological features were evaluated using the Mann-Whitney test. No correlation was observed between expression of OTUD6B-AS1 and age (*p* = 0.57, Figure 2A), gender (*p* = 0.18, Figure 2B), and multifocality (*p* = 0.57, Figure 2C). OTUD6B-AS1 correlated with tumor size (*p* = 0.013, Figure 2D), lymphatic metastasis (*p* = 0.015, Figure 2E), and clinical stage (*p* = 0.0213 Figure 2F).

### OTUD6B-AS1 Inhibits Migration and Invasion of Thyroid Carcinoma

**Figure 3:**
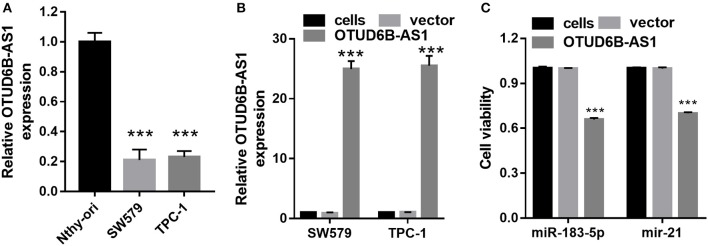
OTUD6B-AS1 inhibits viability of thyroid carcinoma cells. **(A)** The expression of OTUD6B-AS1 in thyroid carcinoma cells SW579 and TPC-1, and normal thyroid cells Nthy-ori were evaluated using qRT-PCR. Data were presented as mean ± sd (*n* = 3). ***p < 0.001, Nthy-ori vs. SW579 or TPC-1. **(B)** The expression of OTUD6B-AS1 in thyroid carcinoma cells SW579 and TPC-1 were evaluated using qRT-PCR after transfected pcDNA3.1-OTUD6B-AS1 (*n* = 3). ***p < 0.001, vector vs. OTUD6B-AS1 group. **(C)** MTT assays of thyroid carcinoma cells (*n* = 3). ***p < 0.001, vector vs. OTUD6B-AS1.

**Figure 4:**
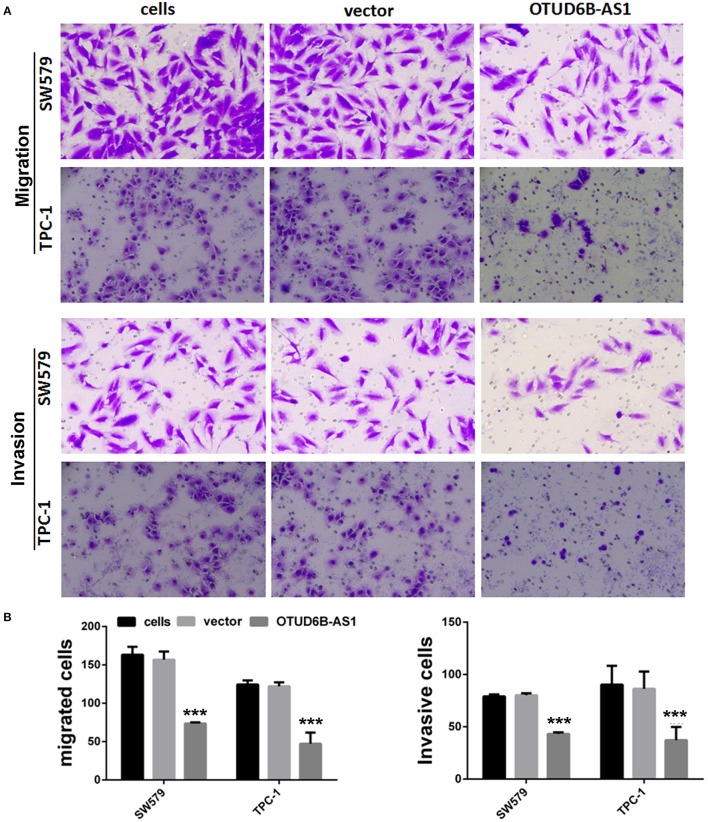
OTUD6B-AS1 inhibits migration and invasion of thyroid carcinoma cells. **(A)** The migration and invasion of thyroid carcinoma cells were evaluated using the transwell assay. **(B)** Statistical analysis of transwell migration and invasion assays. Data were presented as mean ± sd (*n* = 5). ***p < 0.01, vector vs. OTUD6B-AS1.

We first examined the expression of OTUD6B-AS1 in thyroid carcinoma cells SW579 and TPC-1, and normal thyroid cells Nthy-ori. Compared to the normal thyroid cells Nthy-ori, decreased expression of OTUD6B-AS1 was observed in the SW579 and TPC-1 cells (*p* < 0.001, Figure 3A). To elucidate the role of OTUD6B-AS1 in thyroid carcinoma, SW579 and TPC-1 cells were transfected with OTUD6B-AS1–pcDNA3.1 to overexpress OTUD6B-AS1. The qRT-PCR results showed that OTUD6B-AS1 expression was significantly enhanced in the SW579 and TPC-1 cells after transfection with OTUD6B-AS1- pcDNA3.1 compared to transfection with empty pcDNA3.1 (Figure 3B). MTT assay showed overexpression of OTU6B-AS1, and inhibited viability of SW579 and TPC-1 cells (Figure 3C). Transwell migration and invasion assays showed that SW579 and TPC-1 cells overexpressed OTUD6B-AS1 decreased migration and invasion abilities (Figure 4).

### miR-183-5p and miR-21 Were Direct Targets of OTUD6B-AS1

**Figure 5:**
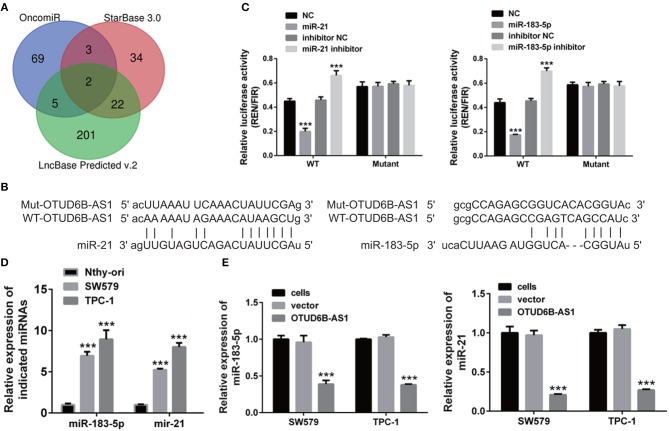
hsa-miR-183-5p and miR-21 was a direct target of OTUD6B-AS1. **(A)** Schematic diagram of screening potential target of OTUD6B-AS1. **(B)** The sequences of the binding sites of lncRNA OTUD6B-AS1 with miR-21 and miR-183-5p. **(C)** luciferase reporter assays of SW579 and TPC-1 cells. psiCHECK-2-WT co-transfected with mimics or inhibitor of miR-183-5p or miR-21 in 293T cells. A scramble RNA (NC) was used as control for mimics of miR-183-5p and miR-21. Inhibitor NC was used as control for inhibitor of miR-183-5p and miR-21. Data were presented as mean ± sd (*n* = 3). ***p < 0.001, NC vs. miR-183-5p or miR-21. Inhibitor NC vs. inhibitor of miR-183-5p or miR-21. **(D)** The expression of miR-183-5p and miR-21 in normal thyroid cells Nthy-ori, and thyroid carcinoma cells SW579, TPC-1 were examined using qRT-PCR. Data were presented as mean ± sd (*n* = 3). ***p < 0.001, Nthy-ori vs. SW579 or TPC-1. **(E)** The ectopic expression of OTUD6B-AS1 caused decreased expression of hsa-miR-183-5p and miR-21 in SW579 and TPC-1 cells. The expression of hsa-miR-183-5p and miR-21 in SW579 and TPC-1 cells were examined using qRT-PCR. U6 was used as internal reference. Data were presented as mean ± sd (*n* = 3). ***p < 0.001, vector vs. OTUD6B-AS1.

Next, we screened potential targets of OTUD6B-AS1 using several online programs, OncomiR, StarBase 3.0 and LncBase Predicted v.2. Using OncomiR, 69 upregulated miRNAs were found. Using StarBase 3.0, 34 miRNAs were found. Using LncBase Predicted v.2, 201 miRNAs were found (Figure 5A). In order to ensure the reliability of the results, we took the overlap of the findings from the three programs. Two miRNAs, miR-183-5p and miR-21, were found to be potential targets of OTUD6B-AS1 (Figure 5A). The sequences of the binding sites of lncRNA OTUD6B-AS1 with miR-21 and miR-183-5p were shown in Figure 5B. In order to verify the predictions of the online programs, luciferase reporter plasmids, psiCHECK-2-WT, containing wild type (WT) OTUD6B-AS1 were created using the psiCHECK-2 vector. psiCHECK-2-WT was co-transfected with miR-183-5p or miR-21 in 293T cells. A scramble RNA (NC) was used as a control for miR-183-5p and miR-21. Decreased luciferase activities were observed in cells co-transfected with psiCHECK-2-WT and its potential target miR-21 or miR-183-5p (Figure 5C). Conversely, increased luciferase activities were observed in cells co-transfected with psiCHECK-2-WT and inhibitors of miR-21 or miR-183-5p (Figure 5C). These data indicated that miR-21 and miR-183-5p are bound to OTUD6B-AS1. In order to determine the binding specificity of miR-21 and miR-183-5p, the binding sites of miR-21 and miR-183-5p were mutated in luciferase reporter plasmids psiCHECK-2-WT, named psiCHECK-2-mutant. No significant differences were observed in cells co-transfected with psiCHECK-2-mutant and miR-21 or miR-183-5p (Figure 5C), nor in cells co-transfected with psiCHECK-2-mutant and miR-21 inhibitor or miR-183-5p (Figure 5C). These data indicate that mutations in the binding sites of miR-21 and miR-183-5p compromised the binding of miR-21 and miR-183-5p to OTUD6B-AS1.

In addition, we examined the expression of miR-183-5p and miR-21 in normal thyroid cells Nthy-ori, and thyroid carcinoma cells SW579 and TPC-1 using qRT-PCR. We found that miR-183-5p and miR-21 were upregulated in the SW579 and TPC-1 cells compared to Nthy-ori cells (Figure 5D). Moreover, decreased expression of miR-183-5p and miR-21 was observed in SW579 and TPC-1 cells that overexpressed OTUD6B-AS1 (Figure 5E). Taken together, these findings support the prediction that miR-21 and miR-183-5p are the direct target of OTUD6B-AS1.

### The Overexpression miR-183-5p and miR-21 Partly Compromised the Inhibitory Effects of OTUD6B-AS1 on Migration and Invasion

**Figure 6:**
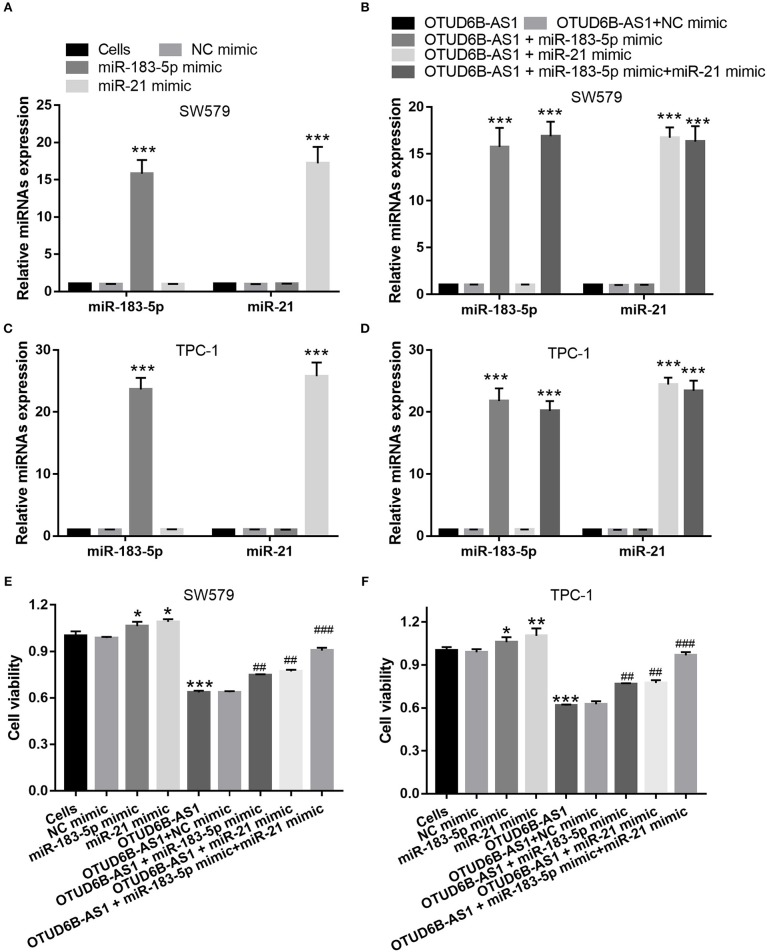
miR-183-5p and miR-21 compromised the inhibitory effects of OTUD6B-AS1 on proliferation of thyroid carcinoma cells. **(A,C)** miR-183-5p or miR-21 expression were measured using qRT-PCR after transfection with miR-183-5p and miR-21 at 48 h in SW579 and TPC-1 cells (*n* = 3). ***p < 0.001, vs. Cells group; **(B,D)** miR-183-5p and miR-21 expression were measured using qRT-PCR after co-transfected OTUD6B-AS1 with miR-183-5p and (or) miR-21 at 48 h in SW579 and TPC-1 cells (*n* = 3). ***p < 0.001, vs. transfection OTUD6B-AS1 group; **(E,F)** MTT assays of thyroid carcinoma cells at 48 h after transfection. Data were presented as mean ± sd (*n* = 3). *p < 0.05, **p < 0.01, and ***p < 0.001, vs. Cells group; ^##^*p* < 0.05 and ^###^*p* < 0.001, vs. OTUD6B-AS1 group.

**Figure 7:**
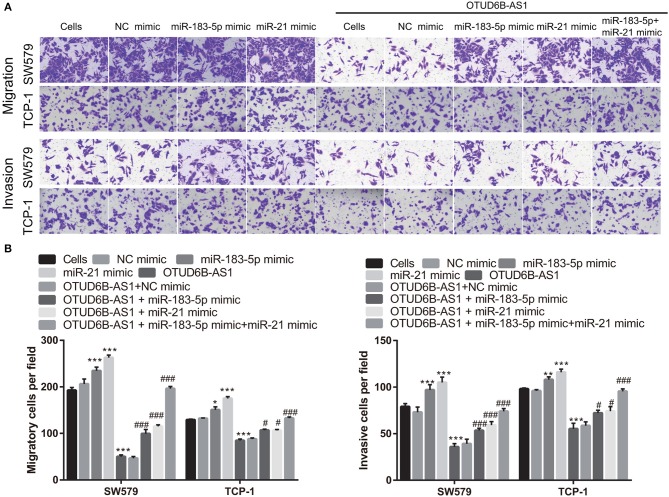
miR-183-5p and miR-21 compromised the inhibitory effects of OTUD6B-AS1 on migration and invasion of thyroid carcinoma cells. **(A)** The migration and invasion of thyroid carcinoma cells were evaluated using the transwell assay at 48 h after transfection. **(B)** Statistical analysis of transwell migration and invasion assays. Data were presented as mean ± sd (*n* = 5). *p < 0.05, **p < 0.01, and ***p < 0.001, vs. Cells group; ^#^*p* < 0.05 and ^###^*p* < 0.001, vs. OTUD6B-AS1 group.

We speculated that OTUD6B-AS1 regulates the migration and invasion of thyroid carcinoma by down-regulating miR-183-5p and miR-21. To verify this speculation, we investigated whether the up-regulation of miR-183-5p and miR-21 can compromise the inhibitory effects of OTUD6B-AS1. The mimics of miR-183-5p and miR-21 were transfected into SW579 and TPC-1 cells. The increased expression of miR-183-5p and miR-21 was verified using qRT-PCR. Compared with Cells group, miR-183-5p and miR-21 expression were significantly increased after transfected miR-183-5p and miR-21 mimic into SW579 and TPC-1 cells (Figures 6A,C). Additionally, compared with OTUD6B-AS1 group, miR-183-5p and miR-21 expression were significantly increased after co-transfected OTUD6B-AS1, miR-183-5p, and (or) miR-21 mimic into SW579 and TPC-1 cells (Figures 6B,D). Compared with Cells group, miR-21 or miR-183-5p overexpression increased the viability of the SW579 and TPC-1 cells (Figures 6E,F). miR-21 or miR-183-5p overexpression also increased the viability of the OTUD6B-AS1-SW579 and TPC-1 cells (Figures 6E,F). Compared with Cells group, miR-21 or miR-183-5p overexpression increased the migration and invasion abilities of the SW579 and TPC-1 cells (Figure 7). Transwell migration and invasion assays also showed that increased migration and invasion abilities were observed in OTUD6B-AS1-SW579 and TPC-1 cells transfected with miR-183-5p and miR-21, compared to those cells transfected with the NC group (Figure 7). Additionally, miR-21 and miR-183-5p overexpression had stacked effect to reverse the effect of OTUD6B-AS1 on the viability, migration, and invasion abilities (Figures 6E,F, 7).

### OTUD6B-AS1-miR-21/miR-183-5p Regulated Downstream Signaling Pathway

Target mRNAs of miR-21 and miR-183-3p was 384 and 509, respectively. KEGG pathway enrichment analysis of 893 target mRNAs were used the DAVID website. We found that OTUD6B-AS1-miR-21/miR-183-5p regulated downstream signaling pathway include MAPK signaling pathway, Regulation of actin cytoskeleton, Wnt signaling pathway, TGF-beta signaling pathway, Adherens junction, Chemokine signaling pathway, and Jak-STAT signaling pathway (Table 3).

**Table 3:** KEGG pathway enrichment analysis of the target mRNAs of miR-21 and miR-183-3p were used DAVID website.

**Term**	**Count**	***P-*value**
MAPK signaling pathway	32	2.44303E-06
Pathways in cancer	36	3.71069E-06
Axon guidance	19	3.19121E-05
Regulation of actin cytoskeleton	24	0.000190054
Wnt signaling pathway	19	0.000255289
GnRH signaling pathway	14	0.000671309
Pancreatic cancer	11	0.00195931
ErbB signaling pathway	12	0.002554004
TGF-beta signaling pathway	12	0.002554004
Insulin signaling pathway	15	0.004613933
Neurotrophin signaling pathway	14	0.005705134
Colorectal cancer	11	0.006122015
Chronic myeloid leukemia	10	0.00867911
Long-term potentiation	9	0.014627969
Melanogenesis	11	0.018570515
Endocytosis	16	0.028079556
Adherens junction	9	0.028937093
Chemokine signaling pathway	16	0.031848154
Jak-STAT signaling pathway	14	0.032298353
Renal cell carcinoma	8	0.04746583
Melanoma	8	0.050618272
Progesterone-mediated oocyte maturation	9	0.050835265
Gap junction	9	0.060018888
Type II diabetes mellitus	6	0.070806025
Oocyte meiosis	10	0.077260826
mTOR signaling pathway	6	0.099472636

## Discussion

The dysregulation of lncRNA expression which acted as oncogenes or tumor-suppressor genes have great significance for the occurrence, development, metastasis, clinical stage and prognosis of thyroid cancer (7–9). OTUD6B-AS1 was first reported in systemic sclerosis (10). It is significantly downregulated in systemic sclerosis skin biopsies (10). In the field of cancer research, Wang and colleague reported OTUD6B-AS1 was downregulated in ccRCC tissue samples, and that patients with low OTUD6B-AS1 expression had shorter overall survival than patients with high OTUD6B-AS1 expression, which showed that the different expression levels of OTUD6B-AS1 indirectly correlated with patient survival (11). We reported downregulation of lncRNA OTUD6B-AS1 in thyroid cancer. The different expression level of OTUD6B-AS1 correlated with tumor size, clinical stage and lymphatic metastasis of thyroid carcinomas. The result suggested that lower OTUD6B-AS1 expression was closely related to the growth and metastasis of thyroid cancer. Consistent results in thyroid cancer and ccRCC indicate down-regulation of OTUD6B-AS1 as important for cancer development.

Under different physiological and pathological conditions, OTUD6B-AS1 is implicated in different cellular biological functions. In systemic sclerosis, OTUD6B-AS1 functions as a regulator of apoptosis (10). OTUD6B-AS1 regulates proliferation and apoptosis via cyclin D1 expression in a sense gene independent manner (10). In tumors, OTUD6B-AS1 regulates proliferation, migration and invasion. It inhibits ccRCC proliferation via the Wnt/beta-catenin signaling pathway (11). In the current study, we reported that OTUD6B-AS1 overexpression inhibited cells viability, migration, and invasion, similar with the effect of OTUD6B-AS1 in ccRCC. The results suggested thatOTUD6B-AS1 acts as a tumor suppressor in thyroid carcinomas. One of the important findings of this study is our identification of two downstream targets of OTUD6B-AS1, miR-183-5p, and miR-21. miR-21 is a well-studied oncogenic miRNA in a large variety of cancers. Up-regulation of miR-21 is implicated in cancer progression and metastasis (22, 23). Several studies indicate that miR-21 expression is associated with the progression and recurrence of thyroid carcinomas (24–26). We showed that miR-21 is a direct target of OTUD6B-AS1. This result found that miR-21 overexpression promoted the viability, migration, and invasion, reversed OTUD6B-AS1 effect in thyroid carcinomas. OTUD6B-AS1 and miR-21 constitute a regulatory axis within thyroid carcinomas. miR-183-5p is another direct target of OTUD6B-AS1. The function of miR-183-5p in cancer is controversial. The role of miR-183 varies in different genetic and pathological contexts. Previous studies indicate that miR-183-5p is an oncogenic miRNA in breast cancer (27), hepatocellular carcinoma (28), colon cancer (29), colorectal cancer (30), bladder cancer (31), and pancreatic cancer (32, 33). There are also several studies indicating that miR-183-5p functions as a tumor suppressor in lung cancer (34), acute myeloid leukemia (35), gastric cancer (36), and osteosarcoma (37). In thyroid carcinomas, miR-183-5p functions as an oncogene and promotes proliferation, migration, and invasion by targeting PDCD4 (38). Our work also found that miR-183-5p overexpression promoted the viability, migration, and invasion, reversed OTUD6B-AS1 effect in thyroid carcinomas, which supports the function of miR-183-5p as an oncogene in thyroid carcinomas. The result also showed that OTUD6B-AS1 inhibits the viability, migration, and invasion of thyroid carcinomas by targeting oncogenic miR-183-5p. OTUD6B-AS1 and miR-183-5p, constitute another regulatory axis of thyroid carcinomas. Additionally, miR-21 and miR-183-5p overexpression had stacked effect to reverse the effect of OTUD6B-AS1 on the viability, migration, and invasion abilities. The result suggested that OTUD6B-AS1-miR-183-5p axis and OTUD6B-AS1-miR-21 axis had coordinated with each other to regulate the viability, migration, and invasion of thyroid carcinomas. We revealed a novel mechanism underlying the inhibitory effect of OTUD6B-AS1 on tumor cells.

Furthermore, OTUD6B-AS1-miR-21/miR-183-5p regulated downstream signaling pathway include MAPK signaling pathway, Regulation of actin cytoskeleton, Wnt signaling pathway, TGF-beta signaling pathway, Adherens junction, Chemokine signaling pathway, and Jak-STAT signaling pathway. Actin cytoskeleton and cell adhere, are closely related to epithelial to mesenchymal transition (EMT) and cell migration (39, 40). Chemokine can recruit macrophage and induced macrophage M2 polarization which promoted tumor lymphatic metastasis (41). MAPK and TGF-beta signaling pathway has been known to mediate cell EMT and migration in thyroid carcinomas (42, 43). Silenced Jak-STAT and Wnt/β-catenin signaling pathway reduced thyroid carcinomas cell viability, migration, and invasion (44, 45). Previous study found that OTUD6B-AS1 silenced Wnt/β-catenin pathway in ccRCC (11). These results suggested that miR-21/miR-183-5p regulated downstream signaling pathway involve the regulation of thyroid carcinomas cell viability, migration, and invasion. However, the current study did not reveal the targets of miR-21 and miR-183-5p and the downstream signaling pathway.

## Conclusions

In conclusion, OTUD6B-AS1 which acts as a tumor suppressor inhibits the viability, migration, and invasion of thyroid carcinomas by targeting miR-183-5p and miR-21. OTUD6B-AS1-miR-183-5p/miR-21 axes constitute a regulatory network within thyroid carcinomas.

## Data Availability Statement

The raw data supporting the conclusions of this article will be made available by the authors, without undue reservation, to any qualified researcher.

## Ethics Statement

This study obtained approval from the Clinical Research Ethics Committee of Xiangya Hospital, Central South University. The patients/participants provided their written informed consent to participate in this study.

## Author Contributions

ZW and XL designed the study. ZW, FX, and TF collected the data. ZW, BJ, WW, and XL analyzed the data. ZW wrote the manuscript. WW and XL revised the manuscript. All authors read and approved the final manuscript.

### Conflict of Interest

The authors declare that the research was conducted in the absence of any commercial or financial relationships that could be construed as a potential conflict of interest.

## References

[1] Zhou P Tian S Li J Zhao Y Liu W Zhang Y. Paradoxes in thyroid carcinoma treatment: analysis of the SEER database 2010-2013. Oncotarget. (2017) 8:345–53. 10.18632/oncotarget.13395 27861148 PMC5352124

[2] Liebner DA Shah MH. Thyroid cancer: pathogenesis and targeted therapy. Ther Adv Endocrinol Metab. (2011) 2:173–95. 10.1177/2042018811419889 23148184 PMC3474640

[3] Mahmoudian-Sani MR Jalali A Jamshidi M Moridi H Alghasi A Shojaeian A. Long non-coding RNAs in thyroid cancer: implications for pathogenesis, diagnosis, and therapy. Oncol Res Treat. (2019) 42:136–42. 10.1159/000495151 30799425

[4] Liu G Zheng J Zhuang L Lv Y Zhu G Pi L. A prognostic 5-lncRNA expression signature for head and neck squamous cell carcinoma. Sci Rep. (2018) 8:15250. 10.1038/s41598-018-33642-1 30323196 PMC6189101

[5] Yan Y Chen X Wang X Zhao Z Hu W Zeng S. The effects and the mechanisms of autophagy on the cancer-associated fibroblasts in cancer. J Exp Clin Cancer Res. (2019) 38:171. 10.1186/s13046-019-1172-5 31014370 PMC6480893

[6] He RZ Luo DX Mo YY. Emerging roles of lncRNAs in the post-transcriptional regulation in cancer. Genes Diseases. (2019) 6:6–15. 10.1016/j.gendis.2019.01.003 30906827 PMC6411652

[7] Li X Zhong W Xu Y Yu B Liu H. Silencing of lncRNA LINC00514 inhibits the malignant behaviors of papillary thyroid cancer through miR-204-3p/CDC23 axis. Biochem Biophys Res Commun. (2019) 508:1145–8. 10.1016/j.bbrc.2018.12.051 30553447

[8] Liu H Deng H Zhao Y Li C Liang Y. LncRNA XIST/miR-34a axis modulates the cell proliferation and tumor growth of thyroid cancer through MET-PI3K-AKT signaling. J Exp Clin Cancer Res. (2018) 37:279. 10.1186/s13046-018-0950-9 30463570 PMC6249781

[9] Zhang K Lv J Peng X Liu J Li C Li J. Down-regulation of DANCR acts as a potential biomarker for papillary thyroid cancer diagnosis. Biosci Rep. (2019) 39:4. 10.1042/bsr20181616 30910839 PMC6470405

[10] Takata M Pachera E Frank-Bertoncelj M Kozlova A Jungel A Whitfield ML. OTUD6B-AS1 might be a novel regulator of apoptosis in systemic sclerosis. Front Immunol. (2019) 10:1100. 10.3389/fimmu.2019.01100 31156645 PMC6533854

[11] Wang G Zhang ZJ Jian WG Liu PH Xue W Wang TD. Novel long noncoding RNA OTUD6B-AS1 indicates poor prognosis and inhibits clear cell renal cell carcinoma proliferation via the Wnt/beta-catenin signaling pathway. Mol Cancer. (2019) 18:15. 10.1186/s12943-019-0942-1 30670025 PMC6341572

[12] Spitschak A Meier C Kowtharapu B Engelmann D Putzer BM. MiR-182 promotes cancer invasion by linking RET oncogene activated NF-kappaB to loss of the HES1/Notch1 regulatory circuit. Mol Cancer. (2017) 16:24. 10.1186/s12943-016-0563-x 28122586 PMC5267421

[13] Pan Y Zhu X Wang K Chen Y. MicroRNA-363-3p suppresses anoikis resistance in human papillary thyroid carcinoma via targeting integrin alpha 6. Acta Biochim Biophys Sin. (2019) 51:807–13. 10.1093/abbs/gmz066 31257410

[14] Salmena L Poliseno L Tay Y Kats L Pandolfi PP. A ceRNA hypothesis: the Rosetta Stone of a hidden RNA language? Cell. (2011) 146:353–8. 10.1016/j.cell.2011.07.014 21802130 PMC3235919

[15] Liu X Fu Q Li S Liang N Li F Li C. LncRNA FOXD2-AS1 functions as a competing endogenous RNA to regulate TERT expression by sponging miR-7-5p in thyroid cancer. Front Endocrinol. (2019) 10:207. 10.3389/fendo.2019.00207 31024447 PMC6463795

[16] Livak KJ Schmittgen TD. Analysis of relative gene expression data using real-time quantitative PCR and the 2(-Delta Delta C(T)) Method. Methods. (2001) 25:402–8. 10.1006/meth.2001.1262 11846609

[17] Wong NW Chen Y Chen S Wang X. OncomiR: an online resource for exploring pan-cancer microRNA dysregulation. Bioinformatics. (2018) 34:713–5. 10.1093/bioinformatics/btx627 29028907 PMC5860608

[18] Li JH Liu S Zhou H Qu LH Yang JH. starBase v2.0: decoding miRNA-ceRNA, miRNA-ncRNA and protein-RNA interaction networks from large-scale CLIP-Seq data. Nucleic Acids Res. (2014) 42:D92–7. 10.1093/nar/gkt1248 24297251 PMC3964941

[19] Paraskevopoulou MD Vlachos IS Karagkouni D Georgakilas G Kanellos I Vergoulis T. DIANA-LncBase v2: indexing microRNA targets on non-coding transcripts. Nucleic Acids Res. (2016) 44:D231–8. 10.1093/nar/gkv1270 26612864 PMC4702897

[20] Agarwal V Bell GW Nam JW Bartel DP. Predicting effective microRNA target sites in mammalian mRNAs. eLife. (2015) 4:5. 10.7554/eLife.05005 26267216 PMC4532895

[21] Huang da W Sherman BT Lempicki RA. Bioinformatics enrichment tools: paths toward the comprehensive functional analysis of large gene lists. Nucleic Acids Res. (2009) 37:1–13. 10.1093/nar/gkn923 19033363 PMC2615629

[22] Xu J Zhang W Lv Q Zhu D. Overexpression of miR-21 promotes the proliferation and migration of cervical cancer cells via the inhibition of PTEN. Oncol Rep. (2015) 33:3108–16. 10.3892/or.2015.3931 25963606

[23] Wang J Chu Y Xu M Zhang X Zhou Y Xu M. miR-21 promotes cell migration and invasion of hepatocellular carcinoma by targeting KLF5. Oncol Lett. (2019) 17:2221–7. 10.3892/ol.2018.9843 30675287 PMC6341730

[24] Guo Z Hardin H Montemayor-Garcia C Asioli S Righi A Maletta F. *In situ* hybridization analysis of miR-146b-5p and miR-21 in thyroid nodules: diagnostic implications. Endocr Pathol. (2015) 26:157–63. 10.1007/s12022-015-9363-x 25771986

[25] Samsonov R Burdakov V Shtam T Radzhabovsmall a CZ Vasilyev D Tsyrlina E. Plasma exosomal miR-21 and miR-181a differentiates follicular from papillary thyroid cancer. Tumour Biol. (2016) 37:12011–21. 10.1007/s13277-016-5065-3 27164936

[26] Sondermann A Andreghetto FM Moulatlet AC da Silva Victor E de Castro MG Nunes FD. MiR-9 and miR-21 as prognostic biomarkers for recurrence in papillary thyroid cancer. Clin Exp Metastasis. (2015) 32:521–30. 10.1007/s10585-015-9724-3 26007293

[27] Macedo T Silva-Oliveira RJ Silva VAO Vidal DO Evangelista AF Marques MMC. Overexpression of mir-183 and mir-494 promotes proliferation and migration in human breast cancer cell lines. Oncol Lett. (2017) 14:1054–60. 10.3892/ol.2017.6265 28693273 PMC5494613

[28] Li ZB Li ZZ Li L Chu HT Jia M. MiR-21 and miR-183 can simultaneously target SOCS6 and modulate growth and invasion of hepatocellular carcinoma (HCC) cells. Eur Rev Med Pharmacol Sci. (2015) 19:3208–17. 26400524

[29] Bi DP Yin CH Zhang XY Yang NN Xu JY. MiR-183 functions as an oncogene by targeting ABCA1 in colon cancer. Oncol Rep. (2016) 35:2873–9. 10.3892/or.2016.4631 26935154

[30] Huangfu L Liang H Wang G Su X Li L Du Z. miR-183 regulates autophagy and apoptosis in colorectal cancer through targeting of UVRAG. Oncotarget. (2016) 7:4735–45. 10.18632/oncotarget.6732 26717041 PMC4826239

[31] Chen D Li SG Chen JY Xiao M. MiR-183 maintains canonical Wnt signaling activity and regulates growth and apoptosis in bladder cancer via targeting AXIN2. Eur Rev Med Pharmacol Sci. (2018) 22:4828–36. 10.26355/eurrev_201808_15618 30070321

[32] Yang X Wang W Zhang X Zou Q Cai L Yu B. Downregulation of miR-183 inhibits the growth of PANC-1 pancreatic cancer cells in vitro and in vivo, and increases chemosensitivity to 5-fluorouracil and gemcitabine. Exp Ther Med. (2019) 17:1697–705. 10.3892/etm.2018.7112 30783438 PMC6364144

[33] Lu YY Zheng JY Liu J Huang CL Zhang W Zeng Y. miR-183 induces cell proliferation, migration, and invasion by regulating PDCD4 expression in the SW1990 pancreatic cancer cell line. Biomed Pharmacother. (2015) 70:151–7. 10.1016/j.biopha.2015.01.016 25776494

[34] Meng F Zhang L. miR-183-5p functions as a tumor suppressor in lung cancer through PIK3CA inhibition. Exp Cell Res. (2019) 374:315–22. 10.1016/j.yexcr.2018.12.003 30528264

[35] Zheng Z Zheng X Zhu Y Gu X Gu W Xie X. miR-183-5p inhibits occurrence and progression of acute myeloid leukemia via targeting erbin. Mol Ther. (2019) 27:542–58. 10.1016/j.ymthe.2019.01.016 30799283 PMC6401194

[36] Cao LL Xie JW Lin Y Zheng CH Li P Wang JB. miR-183 inhibits invasion of gastric cancer by targeting Ezrin. Int J Clin Exp Pathol. (2014) 7:5582–94. 25337200 PMC4203171

[37] Yang X Wang L Wang Q Li L Fu Y Sun J. MiR-183 inhibits osteosarcoma cell growth and invasion by regulating LRP6-Wnt/beta-catenin signaling pathway. Biochem Biophys Res Commun. (2018) 496:1197–203. 10.1016/j.bbrc.2018.01.170 29402412

[38] Wei C Song H Sun X Li D Song J Hua K. miR-183 regulates biological behavior in papillary thyroid carcinoma by targeting the programmed cell death 4. Oncol Rep. (2015) 34:211–20. 10.3892/or.2015.3971 26063221

[39] Bersini S Lytle NK Schulte R Huang L Wahl GM Hetzer MW. Nup93 regulates breast tumor growth by modulating cell proliferation and actin cytoskeleton remodeling. Life Sci Alliance. (2020) 3:623. 10.26508/lsa.201900623 31959624 PMC6971368

[40] Liotta LA. Adhere, degrade, and move: the three-step model of invasion. Cancer Res. (2016) 76:3115–7. 10.1158/0008-5472.can-16-1297 27251085 PMC5547436

[41] Ruytinx P Proost P Van Damme J Struyf S. Chemokine-induced macrophage polarization in inflammatory conditions. Front Immunol. (2018) 9:1930. 10.3389/fimmu.2018.01930 30245686 PMC6137099

[42] Wang DP Tang XZ Liang QK Zeng XJ Yang JB Xu J. Overexpression of long noncoding RNA SLC26A4-AS1 inhibits the epithelial-mesenchymal transition via the MAPK pathway in papillary thyroid carcinoma. J Cell Physiol. (2020) 235:2403–13. 10.1002/jcp.29145 31556116

[43] Bhatti MZ Pan L Wang T Shi P Li L. REGgamma potentiates TGF-beta/Smad signal dependent epithelial-mesenchymal transition in thyroid cancer cells. Cell Signal. (2019) 64:109412. 10.1016/j.cellsig.2019.109412 31491459

[44] Bi CL Zhang YQ Li B Guo M Fu YL. MicroRNA-520a-3p suppresses epithelial-mesenchymal transition, invasion, and migration of papillary thyroid carcinoma cells via the JAK1-mediated JAK/STAT signaling pathway. J Cell Physiol. (2019) 234:4054–67. 10.1002/jcp.27199 30206929

[45] Ma J Huang X Li Z Shen Y Lai J Su Q. FOXE1 supports the tumor promotion of Gli2 on papillary thyroid carcinoma by the Wnt/beta-catenin pathway. J Cell Physiol. (2019) 234:17739–48. 10.1002/jcp.28399 30793770

